# Real‐world outcomes of NSCLC patients receiving tissue or circulating tumor DNA‐guided osimertinib treatment

**DOI:** 10.1002/cam4.2485

**Published:** 2019-08-21

**Authors:** Po‐Lan Su, Szu‐Chun Yang, Yi‐Lin Chen, Yi‐Lin Wu, Chia‐Ying Lin, Wei‐Yuan Chang, Yau‐Lin Tseng, Wu‐Wei Lai, Chung‐Liang Ho, Chien‐Chung Lin, Wu‐Chou Su

**Affiliations:** ^1^ Department of Internal Medicine National Cheng Kung University Hospital College of Medicine National Cheng Kung University Tainan Taiwan; ^2^ Department of Public Health College of Medicine National Cheng Kung University Tainan Taiwan; ^3^ Department of Pathology National Cheng Kung University Hospital College of Medicine National Cheng Kung University Tainan Taiwan; ^4^ Department of Nursing National Cheng Kung University Hospital College of Medicine National Cheng Kung University Tainan Taiwan; ^5^ Department of Diagnostic Radiology National Cheng Kung University Hospital College of Medicine National Cheng Kung University Tainan Taiwan; ^6^ Institute of Clinical Medicine National Cheng Kung University Hospital College of Medicine National Cheng Kung University Tainan Taiwan; ^7^ Department of Surgery National Cheng Kung University Hospital College of Medicine National Cheng Kung University Tainan Taiwan; ^8^ Department of Biochemistry and Molecular Biology College of Medicine National Cheng Kung University Tainan Taiwan; ^9^ Center of Applied Nanomedicine National Cheng Kung University Tainan Taiwan

**Keywords:** circulating tumor DNA, NSCLC, osimertinib, progression‐free survival, T790M mutation

## Abstract

**Background:**

Osimertinib yields significant tumor responses and durations of progression‐free survival (PFS) in patients with acquired T790M mutations. However, the evidence supporting liquid biopsy‐guided treatment is still limited. This study examined the real‐world benefits of osimertinib in patients with tissue or plasma T790M mutations.

**Methods:**

From January 2016 to June 2018, a total of 183 non‐small‐cell lung cancer patients were enrolled. The presence of the T790M mutation was assessed by either tissue or plasma. The PFS, overall survival, and tumor response rates of the patients were calculated and compared with those of previous clinical trials.

**Results:**

T790M mutations were detected in 51.5% of the patients, including 64 of 140 (45.7%) who underwent liquid biopsies and 23 of 29 (79.3%) who underwent tumor biopsies. After excluding those in clinical trials, 46 patients received osimertinib, including 33 with positive plasma and 13 with positive tissue results for T790M mutations. The median PFS was 11.3 months (interquartile range: 5.2‐NR) in all the T790M‐positive patients and 10.1 months (interquartile range: 5.9‐NR) in the plasma T790M‐positive patients. The overall survival, meanwhile, was not reached, whereas the one‐year survival rate was 66.1% in all the patients and 61.4% in those who were plasma T790M‐positive. The objective response rate and disease control rate were 37.8% and 91.9% in all the patients and 34.6% and 92.3% in the plasma T790M‐positive group, respectively. Using a Cox proportional hazards regression, we determined that male gender was a poor prognostic factor for PFS.

**Conclusions:**

In this retrospective real‐world analysis, it was determined that both tissue and plasma T790M mutations can be used to guide treatment with osimertinib. Similar disease control rates and survival durations were observed in comparison to those of phase 3 clinical trials.

## Graphical abstract

This study examined the real‐world benefits of osimertinib in patients with tissue or plasma T790M mutations. The progression‐free survival, overall survival, and tumor response rates of 46 non‐small‐cell lung cancer patients were calculated and compared with those of previous clinical trials. It was determined that both tissue and plasma T790M mutations can be used to guide treatment with osimertinib.

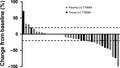

## 1 BACKGROUND

Epidermal growth factor receptor (EGFR) mutations are key genetic drivers of non‐small‐cell lung cancer (NSCLC) and are present in about 10% of the Caucasian population and 40%‐50% of the Asian population.^1^ The response rates to first‐generation EGFR tyrosine kinase inhibitors (TKIs) range between 56% and 74%, and the median progression‐free survival (PFS) durations range from 9 to 13 months.^2^ However, most patients experience disease progression, and about 50%‐70% of them develop newly acquired resistance EGFR p.Thr790Met (T790M) point mutations.^3^, ^4^, ^5^, ^6^, ^7^ These acquired mutations enhance the binding affinity of adenosine triphosphate on the EGFR kinase domain and, in turn, decrease the efficacy of first‐ and second‐generation EGFR‐TKIs. Osimertinib, a third‐generation inhibitor, was designed to and has been proven to selectively target T790M.^8^, ^9^, ^10^ Previous clinical trials have shown the promising efficacy of this third‐generation EGFR‐TKI in patients who experience disease progression after treatment with first‐ and second‐generation EGFR‐TKIs.^9^, ^10^, ^11^ A phase 3 clinical trial (AURA 3) also reported enhanced PFS associated with osimertinib in comparison to standard chemotherapy in NSCLC patients with an acquired T790M mutation.^12^

In order to use osimertinib clinically in a patient, that patient must be shown to have the T790M mutation in his or her tissue. However, tumor rebiopsy continues to present challenges, especially in patients with poor performance status and tumor inaccessibility. For these reasons, researchers have developed a number of liquid biopsy platforms that can be utilized as complements to or in lieu of tissue biopsy.^13^, ^14^, ^15^ With the advancement of genotyping assays—namely, droplet digital polymerase chain reactions (ddPCRs) and beads, emulsions, amplification, and magnetics (BEAMing) digital polymerase chain reaction (dPCR)—the mutation status of a patient can be detected using cell‐free plasma DNA.^5^, ^16^, ^17^, ^18^, ^19^ However, studies on the efficacy of osimertinib using plasma testing remain scarce and limited in terms of patient numbers.^20^ In this study, therefore, we evaluated the feasibility of performing T790M identification through circulating tumor DNA (ctDNA) in blood samples among EGFR‐mutant NSCLC patients exhibiting disease progression after treatment with first‐ or second‐generation EGFR‐TKIs. We also retrospectively evaluated the efficacy of osimertinib in plasma and tissue T790M‐positive patients.

## 2 METHODS

### 2.1 Patients

From January 2016 to June 2018, patients with advanced EGFR‐mutated NSCLC who experienced radiological or clinical progression after treatment with one or more first‐ or second‐generation EGFR‐TKIs were recruited; that is, no maximum limit was set for the number of previous EGFR inhibitors or systemic therapies a patient had received. All of the patients were positive for EGFR mutation at their initial diagnoses. Upon disease progression, genotyping detection based on tissue, liquid, or both was performed based on the clinical judgment of the treating physician. The detection of the EGFR T790M mutation in cell‐free plasma DNA was conducted in the Department of Pathology of National Cheng Kung University Hospital. When a positive result for the T790M mutation was found in a tissue or liquid biopsy, 80 mg per day of osimertinib was prescribed through the compassionate‐use programs of Astrazeneca until the occurrence of disease progression or the appearance of unacceptable adverse effects. The study protocol was approved by the institutional ethics committee of National Cheng Kung University Hospital (IRB number: A‐ER‐106‐205).

### 2.2 Outcomes

All the patients received a chest computed tomography scan before the initiation of the osimertinib treatment and every 12 weeks thereafter to evaluate their tumor responses. Brain imaging and bone scans were performed if there were related symptoms. The primary endpoint was PFS. The secondary endpoints included the disease control rate, overall response rate, overall survival (OS), and the percentage of T790M mutation‐positive patients identified using liquid biopsy. PFS was calculated from the date of osimertinib initiation until the date of radiological progression according to the Response Evaluation Criteria in Solid Tumors (RECIST) v1.1^21^ or death, with censoring at the date of the last follow‐up in the event that the patient had not progressed. The overall response rate was calculated as the percentage of patients who exhibited a partial response or complete response in the first image study after the initiation of the osimertinib treatment, while the disease control rate was defined as the percentage of patients who exhibited a partial response, complete response, or stable disease. Furthermore, the duration of OS was defined as the period from the initiation date of osimertinib treatment until the date of death.

### 2.3 Tumor mutation analysis

To perform the EGFR mutation analysis, tumor tissues were collected from the primary lung cancer or metastatic lesions, with tissue samples having a tumor content of more than 80% as determined using microscopy with hematoxylin and eosin staining being selected for the analysis. DNA was extracted using a QIAamp DNA FFPE tissue kit (Qiagen) eluted in ATE (QIAmp Tissue Elution) buffer in combination with the QIAcube automated extractor (Qiagen) according to the manufacturer's instructions. The EGFR PCR Kit (EGFR RUO Kit) and the therascreen EGFR RGQ PCR Kit (EGFR IVD Kit, Qiagen) were used to identify mutated EGFR DNA. These kits utilized a combination of Scorpions real‐time PCR technology and amplification‐refractory mutation system (ARMS) technology for the detection of the mutations with real‐time quantitative PCR.^22^

### 2.4 Cell‐free DNA extraction

Cell‐free DNA was isolated from 1 mL of plasma with the QIAamp Circulating Nucleic Acid kit (Qiagen) according to the manufacturer's instructions. Briefly, samples were centrifuged at 1000 *g* for 2 minutes, and the resulting supernatant was then transferred to a clean tube before the DNA extraction. Subsequently, lysate buffer (ACL) (800 μL) and carrier RNA (5 μL) were added, and the supernatant was incubated at 60°C. After supplementing the mixture with binding buffer (ACB), the mixture was transferred to ice for 5 minutes, treated with QIAvacuum, and washed with buffer. The eluted cell‐free DNA was stored at −80°C until analysis.

### 2.5 In‐house T790M detection method

The test was performed in duplicate on samples containing 21 µL of Super Therm Gold Master Mix (Bionovas Biotechnology), 2 µL of T790M primers (10 µmol/L), 2 µL of control primers (2.5 µmol/L), and 2 µL of working DNA (15 ng/µL) at a final volume of 27 µL. The following conditions were utilized in performing the PCR assay: 5 minutes at 95°C, followed by 35 cycles of 30 seconds at 95°C, 30 seconds at 63°C, 30 seconds at 72°C, and 20 seconds at 73°C. The human AF4 gene primers (exon 3 and exon 11; GenBank accession no. Z83687) were amplified products of 100 bp used to indicate the quality of the PCR reaction.^23^ The expected PCR product sizes of T790M and the control alleles were 150 bp and 100 bp, respectively.

Analysis of the PCR products was conducted using high‐resolution capillary electrophoresis with the QIAxcel^®^ DNA high‐resolution gel cartridge (Qiagen, Hilden, Germany) on the QIAxcel system (Qiagen) according to the manufacturer's instructions. The sizes of the PCR products were calculated, and the genotypes were determined using Qiagen BioCalculator^®^ software. The DNA size markers (Qiagen) ranged from 50 to 800 bp. The alignment marker (Qiagen) consisting of 15 bp and 1000 bp fragments corresponded to a 15‐second sample injection time at 5 kV and a 420‐second separation time at 5 kV.

### 2.6 Statistical analysis

The clinical characteristics of the patients, including, sex, age, the presence or absence of brain metastasis, tissue genotyping at initial diagnosis, and preceding EGFR‐TKIs, were recorded. The Kaplan–Meier method was used to estimate the PFS and OS. A Cox proportional hazards regression was also performed to evaluate the determinants of PFS and OS. Age, sex, tumor size, nodal stage, EGFR mutation subtypes, and sites of disease progression were chosen as the prognostic factors, and the statistical analysis was carried out with SAS version 9.4 (SAS Institute). All the reported *P*‐values were two‐sided.

## 3 RESULTS

### 3.1 Patient characteristics and T790M mutation rate

**Figure 1:**
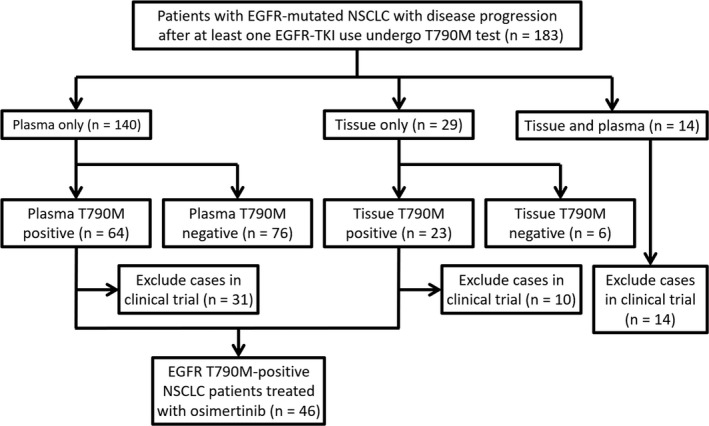
Flow chart for patient enrollment. (EGFR, epidermal growth factor receptor; NSCLC, non‐small‐cell lung cancer; TKI, tyrosine kinase inhibitor)

Of the total of 248 patients with advanced EGFR‐mutated NSCLC recruited from January 2016 to June 2018, 183 underwent examinations for T790M. Among these patients, 140 and 29 patients received liquid and tissue biopsies, respectively, while 14 of the patients received both types of biopsies. Figure 1 shows the flow chart for enrolling the subjects. T790M mutations were detected in 51.5% of the patients who received only one type of biopsy, including 64 of the 140 (45.7%) who underwent liquid biopsies and 23 of the 29 (79.3%) who underwent tumor biopsies. Among the patients who had received only one EGFR‐TKI therapy before receiving osimertinib, the rates for positive T790M results were 62% (47/76), 44% (14/32), and 45% (20/44) in patients receiving gefitinib, erlotinib, and afatinib, respectively. There were no significant differences in these rates of positive results between the patients who had received the different EGFR‐TKIs (*P* = .11). After excluding patients in clinical trials, a total of 46 T790M‐positive patients were enrolled in the study.

The baseline characteristics of the T790M‐positive patients are summarized in Table 1. All the patients had adenocarcinoma histology and were at the advanced stage. The mean age of the patients was 61.8 (range 52.9‐75.2) years, and there were 13 male patients (28.3%) and 33 female patients (71.7%). Fourteen (30.4%) patients had brain metastases. The EGFR genotyping at the initial diagnosis showed Del 19 mutations in 19 (41.3%), L858R mutations in 22 (47.8%), and rare mutations in five (10.9%) of these NSCLC patients. All the patients were pretreated with EGFR‐TKIs: 25 (54.3%), 14 (30.4%), and 7 (15.2%) received gefitinib, erlotinib, and afatinib, respectively.

**Table 1:** Baseline characteristics of T790M‐mutated patients

Characteristic	No. of patients (%) N = 46
Age	
Median (range), (y)	62 (53‐74)
<60 y	21 (45.8%)
>60 y	25 (54.2%)
Sex	
Female	33 (71.7%)
Male	13 (28.3%)
Brain metastases	14 (30.4%)
EGFR mutation	
Exon 19 deletion	19 (41.3%)
L858R	22 (47.8%)
Others	5 (10.9%)
First‐line EGFR‐TKIs	
Gefitinib	25 (54.3%)
Erlotinib	7 (15.2%)
Afatinib	14 (30.4%)

Abbreviations: EGFR, epidermal growth factor receptor; TKI, tyrosine kinase inhibitor.

### 3.2 Response rate of osimertinib

**Figure 2:**
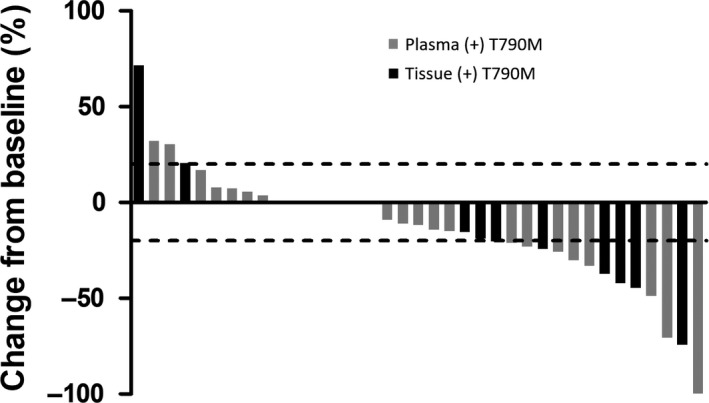
Tumor responses with osimertinib in plasma (gray bar) and tissue (black bar) T790M‐positive patients

Of the 46 patients with tissue‐ or plasma‐positive T790M NSCLC, 37 had measurable target lesions that were evaluated for responses. Among the 9 patients without measurable target lesions, six had pleural effusion only, and three deteriorated rapidly. These nine patients were also enrolled in the study. Of the 37 other patients, one had a complete response, 13 had partial responses, 20 had stable diseases, and four experienced disease progression. The overall response rate was 37.8%, and the disease control rate was 91.9% (Figure 2). In the 26 patients with plasma‐positive T790M and measurable lesions, one had a complete response, eight had partial responses, 15 had stable diseases, and two experienced disease progression. The disease control rate was 92.3%, while the objective response rate was calculated to be 34.6%.

### 3.3 Progression‐free survival and overall survival

**Figure 3:**
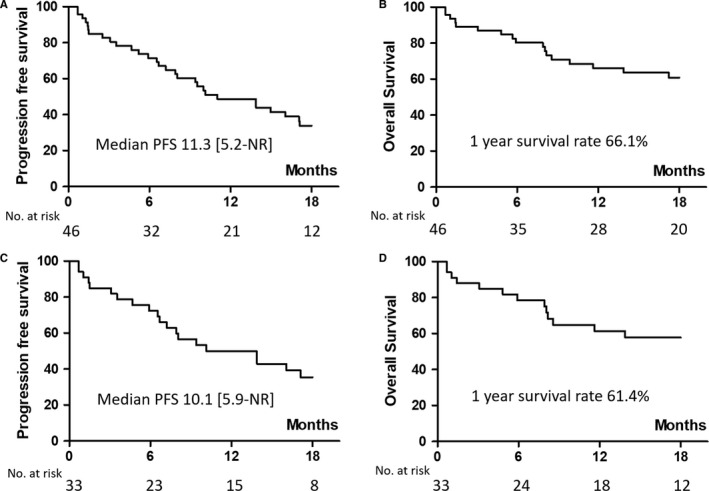
A, PFS in all patients treated with osimertinib. (B), OS in all patients treated with osimertinib. (C), PFS in patients treated with osimertinib according to positive plasma T790M. (D), OS in patients treated with osimertinib according to positive plasma T790M. PFS, progression‐free survival; OS, overall survival

After the median follow‐up period of 17.1 months, the median PFS of all 46 patients was 11.3 months (interquartile range, 5.2 to NR); however, the median OS was not reached (Figure 3A,B). Using a Cox proportional hazards regression, we determined that male gender was a poor prognostic factor for PFS (Table 2). In the subgroup of patients with positive plasma T790M, the median PFS was 10.1 months (interquartile range, 5.9 to NR), but the median OS also was not reached (Figure 3C,D). The median PFS durations of the patients receiving osimertinib immediately after a first‐line EGFR‐TKI and later than a third‐line treatment were 11.0 months (interquartile range, 2.5 to NR) and 13.9 months (interquartile range, 6.7 to NR), respectively (Figure S1A), while the median OS in both groups was not reached (Figure S1B). There were no significant between‐group differences for the PFS and OS groups (log rank *P* = .781 and .335, respectively).

**Table 2:** Cox proportional hazards regression results for progression‐free survival and overall survival

		Progression‐free survival		Overall survival	
		HR (95% CI)	*P* value	HR (95% CI)	*P* value
Age	>65 versus <65	2.23 (0.83‐6.01)	.114	3.15 (0.71‐14.03)	.132
Sex	Male versus female	2.73 (1.03‐7.24)	.043	4.27 (0.96‐18.95)	.056
Tumor size	>3 cm versus <3 cm	1.67 (0.65‐4.27)	.288	1.24 (0.30‐5.07)	.766
Nodal involvement	N2 versus N1	0.54 (0.12‐2.52)	.431	0.25 (0.02‐4.08)	.333
	N3 versus N1	2.92 (0.99‐8.62)	.052	3.85 (0.69‐21.35)	.123
EGFR mutation	Del 19 versus others	0.51 (0.19‐1.38)	.186	0.70 (0.17‐2.80)	.609
Sequential	Second‐line versus ≥third‐line cohorts	0.64 (0.25‐1.66)	.360	0.86 (0.22‐3.32)	.828
Disease progression	Intracranial versus extracranial	0.57 (0.17‐1.92)	.362	0.36 (0.07‐1.98)	.242

Abbreviation: EGFR, epidermal growth factor receptor.

## 4 DISCUSSION

In comparison to liquid biopsies, tissue biopsies have many limitations. Patients may suffer from complications after rebiopsy, such as pneumothorax, which is reported to occur in 17%‐27% of patients.^24^ Also, about 25% of cases provide too few tumor cells or none at all, such that an EGFR mutation assay cannot be conducted.^25^ A single biopsy specimen may miss cancer heterogeneity, but plasma ctDNA can potentially capture it.^26^, ^27^ Many technologies have been developed to improve the sensitivity of tests used to detect genetic mutations in ctDNA. We developed an in‐house method based on an ARMS and compared our results to those of four well‐established technologies, namely, the Bio‐Rad ddPCR system, PANAMutype, the therascreen EGFR Plasma RGQ PCR Kit, and the Cobas EGFR Mutation kit. The clinical performance was analyzed in a clinical cohort of 46 EGFR‐mutated NSCLC patients with acquired EGFR‐TKI resistance. It was found that the detection rate of our platform was equivalent to those of the other four platforms (manuscripts in submission). We employed a straightforward ARMS strategy that combined a higher melting temperature with high‐resolution capillary electrophoresis to enhance the specificity and sensitivity of T790M detection in ctDNA. In two recent prospective studies using a ctDNA‐guided osimertinib treatment, the positivity of plasma T790M was enhanced by 60% using tagged amplicon sequencing and by 71% using dPCR.^20^, ^28^ The detection rate of T790M mutations in liquid biopsies from EGFR‐mutant patients progressing after treatment with an EGFR‐TKI in the current study was 45.7%, which was only slightly lower than the rates for the two studies referenced above, while the detection rates for other retrospective studies have ranged from 50% to 70%.^5^, ^20^, ^28^, ^29^, ^30^ Genotyping using a plasma sample is minimally invasive and has thus become an attractive alternative method for T790M detection.

The AURA 3 phase III trial revealed that osimertinib has efficacy higher than that of platinum plus pemetrexed in T790M‐mutated advanced NSCLC patients who have experienced disease progression while receiving first‐line EGFR‐TKI therapy, and osimertinib has become a standard treatment for these patients.^12^ Before treatment with osimertinib can be commenced, it is required that the presence of a T790M mutation in tumor cells be established by either tissue or plasma genotyping.^12^, ^20^, ^28^ Recently, a review conducted jointly by American Society of Clinical Oncology (ASCO) and the College of American Pathologists proposed that the evidence for the clinical validity and utility of the majority of ctDNA assays for advanced cancer is insufficient. However, a growing range of new NGS platforms have been developed in recent years.^31^, ^32^, ^33^ Moreover, the International Association for the Study of Lung Cancer recently issued a consensus statement holding that liquid biopsies are reliable and should be considered as surrogates for a tissue biopsy in the event that a tissue biopsy cannot be performed, especially with respect to the detection of T790M mutations.^34^ Nonetheless, there is still no definite conclusion regarding the use of liquid biopsies as surrogates for tissue biopsies because some studies have reported that the level of validity needed for widespread use in routine clinical diagnostics has not yet been achieved for liquid biopsies.^35^

There have been a limited number of trials in which the outcomes of treatment for a targeted therapy that was selected solely based on the result of a ctDNA assay were prospectively tested. Oxnard et al conducted a retrospective analysis comparing osimertinib efficacy guided by either plasma or tissue genotyping for advanced EGFR‐mutant NSCLC.^5^ In that study, the reported ORR and median PFS results for the patients with T790M‐positive plasma (ORR, 63%; PFS, 9.7 months) and the patients with T790M‐positive tumor (ORR, 62%; PFS, 9.7 months) samples were similar. The first prospective study investigating the efficacy of osimertinib guided by plasma genotyping was conducted by Remon et al Among 16 patients with positive plasma T790M and evaluable lesions, the ORR was 62.5% and the rate of disease control was 100%. The 1‐year PFS, meanwhile, was as high as 52%.^20^ However, the number of patients was relatively low, and patients with T790M‐positive tumors were not enrolled. Despite the fact that plasma genotyping has many advantages, its low sensitivity and high false‐negative rate are major concerns.^36^ In Oxnard's study,^5^ the ORR of patients with T790M‐negative plasma was found to be 46%, and the PFS was 8.2 months. The tumor genotyping was successful in distinguishing between a subset of patients who were negative for T790M and had poor outcomes (ORR, 25%; PFS, 2.8 months) and another subset of patients who were positive for T790M and had better outcomes (ORR, 69%; PFS, 16.5 months). Based on these findings, the paradigm for the use of plasma genotyping for T790M proposed by several experts emphasizes that if the plasma genotyping is negative for T790M, then tissue genotyping should be performed.^5^, ^34^, ^37^, ^38^ Following this algorithm, Buder et al conducted a clinical trial to evaluate osimertinib response. In that study, the objective response rate was found to be 70% while the disease control rate was 81% in patients with positive plasma T790M as determined using ddPCR or by tissue analyses if they were plasma T790‐negative. However, in real‐world settings, the detection of T790M based on the analysis of cell‐free plasma DNA or supported by tissue biopsy as determined by the treating physician may be an alternative choice for guiding treatment with osimertinib.

Of the various types of studies, randomized controlled trials (RCTs) provide the strongest possible form of evidence, and their results are usually the bases for the decision‐making guidelines used in clinical practice. However, RCTs do have some drawbacks, including the underrepresentation of vulnerable patient groups, a lack of long‐term safety data, and limited generalizability. For example, a variety of patient groups who do not meet the strict criteria for inclusion in RCTs are seen in clinical practice. As such, real‐world data from observational studies or databases can be helpful for answering a wide spectrum of clinical questions and addressing the drawbacks of clinical trials. The present study constitutes the first real‐world study suggesting that T790M detection via cell‐free plasma DNA analysis or supported by the results of a tissue biopsy can be used to guide treatment with osimertinib. In the AURA 3 study trial,^12^ patients with T790M‐positive tumors had a disease control rate of 93% and a median PFS of 9.6 months. In the current study, we confirmed that treatment guided by plasma or tissue T790M mutations could result in a similar median PFS (11.3 months) and disease control rate (91.9%). In patients positive for T790M in plasma, the disease control rate was 92.3%, and the median PFS was 10.1 months. Though the OS data from the Aura 3 study are not available, the updated results from a pooled analysis of two Phase II studies (namely, the AURA extension and AURA2 studies) indicated that there was no significant difference between the second‐line and ≥third‐line cohorts, who received 80 mg of osimertinib once daily, in terms of median OS (95% CI, 25.8 versus 24.0). The 12‐month and 24‐month survival rates were 80% and 56%, respectively.^39^ In this study, we found that the 1‐year OS rate was 66.1%, which was slightly lower than that found in a previous report. We further conducted a Cox proportional hazards regression to identify the prognostic factors that would indicate when patients could benefit from receiving osimertinib in a real‐world setting. Similar to the results for the study referenced above, our results for this study indicated that the sequential use of chemotherapy and osimertinib did not affect survival (Table 2 and Figure S1). Also, the observation that intracranial progress after the failure of previous treatment did not affect the OS implied good penetration of osimertinib into the CNS. However, male patients tended to have poor prognoses. Although male gender was previously found to be a poor prognostic factor in patients receiving first‐line EGFR‐TKI,^40^ the significance of gender in patients receiving osimertinib has not been previously reported. In the AURA 3 study, male patients tended to have a higher hazard ratio of PFS after receiving osimertinib compared to chemotherapy as compared to female patients (0.43 versus 0.34), but that difference was not statistically significant.

This study had several limitations that should be acknowledged. First, the present study was a retrospective study of patients seen at a single center, and any patients taking part in clinical trials were excluded. However, the patients who joined the clinical trials also had positive results for tissue T790M, and if they had been included in the analyses, the response rate would not have been so low. In contrast to a review article,^41^ our real‐world data did not show a substantial difference from the results of clinical trials.^5^, ^20^, ^28^ Second, the sensitivity and specificity of the liquid biopsies could not be examined since most of the patients did not concomitantly receive both tissue and liquid biopsies. Given this lack of tissue biopsies for comparison, differences in the PFS and OS in the patients could not be assessed, which potentially biased the results. A prospective study comparing the survival rates of two such groups of patients would be worthwhile in the future. Finally, it is not clear whether the results of this study can be extrapolated to various other ctDNA platforms. A larger cohort study comparing more methods, including dPCR, BEAMing PCR, and next‐generation sequencing, would also be warranted in the future to guide the selection of patients who would benefit from osimertinib use.

## 5 CONCLUSIONS

We verified the role of plasma genotyping and tissue genotyping in guiding osimertinib treatment in the real world. Moreover, the PFS, objective response rate, and disease control rates found in the present study were similar to those found in previous trials. Overall, the study results indicate that both plasma genotyping and tissue genotyping for T790M detection can be used to predict the response to osimertinib in a real‐world setting.

## AUTHOR CONTRIBUTIONS

Po‐Lan Su, Szu‐Chun Yang and Yi‐Lin Chen contributed equally to this work. PLS, SCY, YLW, CLH, and CCL conceived and designed the experiments. PLS, SCY, YLW, CCL, WYC, YLT, WWL, and WCS contributed materials. PLS, SCY, YLW, and CCL analyzed and interpreted the patient data. YLC and CLH performed the histological and genetic examinations. CYL analyzed the radiologic data. PLS, SCY, YLC, and CCL were major contributors to the writing of the manuscript. All the authors read and approved the final manuscript.

## ETHICS APPROVAL AND CONSENT TO PARTICIPATE

The study protocol was approved by the institutional ethics committee of National Cheng Kung University Hospital (IRB number: A‐ER‐106‐205). Consent to participate was sought from all participants.

## COMPETING INTERESTS

The authors declare that they have no competing interests.

## Data Availability

The datasets for this manuscript are not publicly available due to legal restrictions imposed by the government of Taiwan in relation to the “Personal Information Protection Act.” Requests for access to the datasets should be directed to Chien‐Chung Lin, MD, PhD.
