# Supplementary figures and images for: Real‐world outcomes of NSCLC patients receiving tissue or circulating tumor DNA‐guided osimertinib treatment
Source: Cancer Med. 2019 Aug 21;8(13):5939–47. doi: 10.1002/cam4.2485 (PMC6792511; doi:10.1002/cam4.2485)

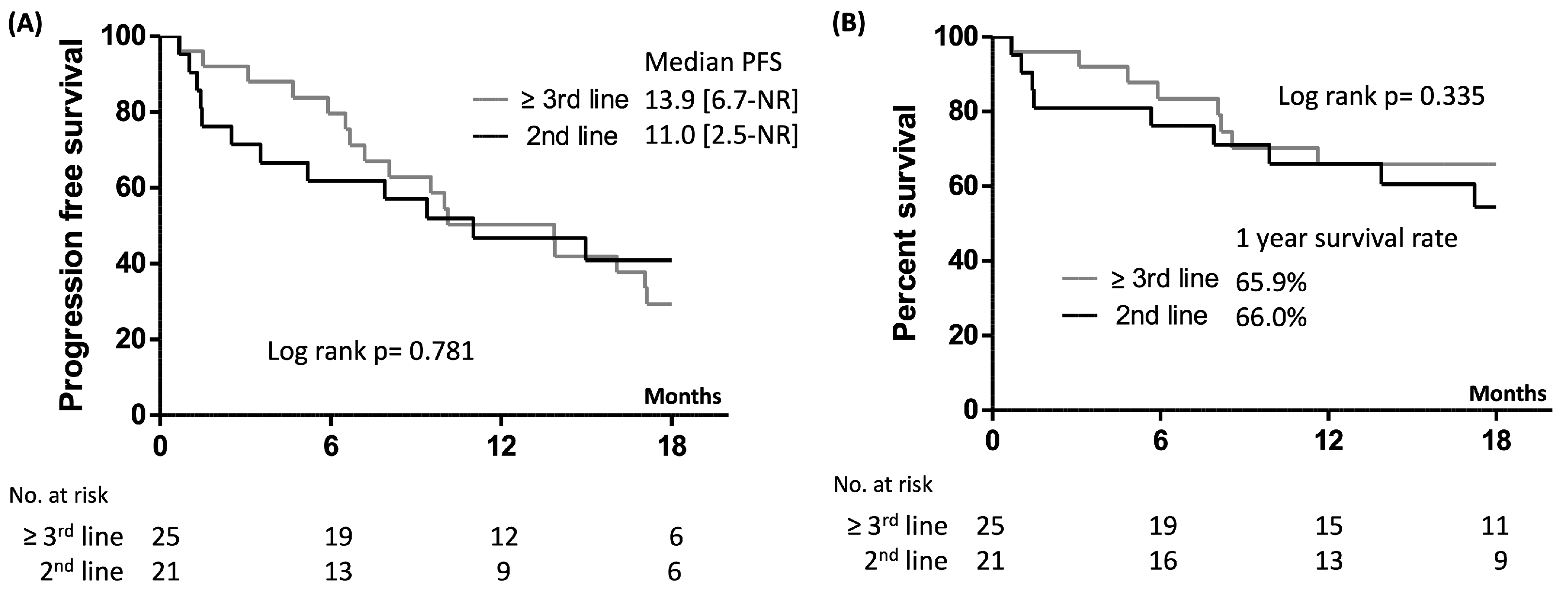

Supplement: Supplementary file 1 [file CAM4-8-5939-s001.tif]
